# How Athila retrotransposons survive in the *Arabidopsis *genome

**DOI:** 10.1186/1471-2164-9-219

**Published:** 2008-05-14

**Authors:** Antonio Marco, Ignacio Marín

**Affiliations:** 1 Center for Evolutionary Functional Genomics, The Biodesign Institute, Arizona State University, USA; 2 Instituto de Biomedicina de Valencia (CSIC), Valencia, Spain

## Abstract

**Background:**

Transposable elements are selfish genetic sequences which only occasionally provide useful functions to their host species. In addition, models of mobile element evolution assume a second type of selfishness: elements of different familes do not cooperate, but they independently fight for their survival in the host genome.

**Results:**

We show that recombination events among distantly related Athila retrotransposons have led to the generation of new Athila lineages. Their pattern of diversification suggests that Athila elements survive in *Arabidopsis *by a combination of selfish replication and of amplification of highly diverged copies with coding potential. Many Athila elements are non-autonomous but still conserve intact open reading frames which are under the effect of negative, purifying natural selection.

**Conclusion:**

The evolution of these mobile elements is far more complex than hitherto assumed. Strict selfish replication does not explain all the patterns observed.

## Background

Mobile elements are selfish genomic parasites that only rarely benefit their hosts [1-4]. They belong to two main classes, with or without RNA intermediates, and most eukaryotic genomes contain several types or *families *of elements of each class [5-7]. A family is a set of very similar sequences that generally include some active elements plus a variable number of non-autonomous, defective copies derived from the active ones. Classical mobile element evolution models suggested that selfishness drives the evolution of each family. Altruistically amplifying either defective copies or elements of other families would decrease the likelihood of long-term survival for a family of elements [5,6,8]. The available data for the *Saccharomyces cerevisiae *and *Drosophila melanogaster *genomes, among others, in which the rule is to find families of recent origin, composed by almost identical and highly active elements [9,10], agrees well with those models. However, whether elements that pervade other genomes, especially those with larger amounts of repetitive sequences, follow the same dynamics has been less extensively studied. In fact, the replication of some types of non-autonomous sequences (e. g. SINEs, MITEs, probably several types of retrotransposon-derived plant repeats) present in large numbers in some genomes depend on mobile elements (reviewed in [11-13]). It is not obvious what kind of advantage may obtain the mobile elements involved, and therefore those non-autonomous sequences are considered to replicate parasitically. However, it is possible to envisage situations in which the amplification of non-autonomous elements contributes to the survival of active elements, a possibility that remains largely unexplored. Some evidence for such type of cooperation within a family is available. For example, active *Drosophila *P elements may improve their likelihood of survival by replicating particular types of defective elements that negatively control the transposition rates of the active ones, thus diminishing the harmful effects on the host (reviewed in [14]; see also [15] for related examples).

Athila is one of the best-known plant long-terminal-repeat (LTR) retrotransposons [16-20]. It belongs to the Ty3/Gypsy group, evolutionary closely related to mammalian retroviruses [21]. Actually, some Athila retrotransposons and a few related plant elements are structurally identical to simple retroviruses. They have, in addition to their *gag *and *pol *genes, a third ORF, generally absent in other LTR retrotransposons. It may encode an envelope (Env) protein, potentially able to allow the generation of viral infective particles ([17,19,20]; see review [22]). However, whether Athila behaves as an infective retrovirus is still unknown. The evolution of Athila retrotransposons has been traced back using phylogenetic analyses based on their reverse transcriptase (RT) sequences, which are part of the *pol *gene [17,18,20,22,23]. These analyses demonstrated that Athila elements are highly heterogenous. Particularly, our group showed that Athila RTs are more variable than those of other eight lineages of *Arabidopsis *Ty3/Gypsy retrotransposons and that there is no relationship between the degree of similarity among elements and the pattern of presence or absence of *env *sequences, suggesting that Athila evolution follows a complex pattern [18].

In this study, we show that the combined analyses of Athila *gag*, *env *and *pol *sequences provides a novel view of the evolutionary forces acting on these retrotransposons in the *Arabidopsis *genome. We determine that most Athila elements lack *pol *sequences and therefore are non-autonomous. Some of these elements have however retained intact ORFs that encode for Gag and Env proteins. These ORFs are under the effect of negative, purifying selection and therefore they must be functional. Moreover, diversification and survival of Athila elements in *Arabidopsis *has often involved recombination among distantly-related elements. In one particular case, recombination involving non-autonomous elements has contributed to generate an active element that moreover has acquired a typical retroviral structure. These results are not compatible with the simplistic view of selfish amplification of independent Athila families.

## Results

### *Arabidopsis *Athila elements can be divided into ancient families, many of them exclusively composed by non-autonomous elements

As already indicated above, the evolutionary analyses performed so far on Athila elements have been focused on comparing RT sequences. However, when we deeply examined the diversity of Athila elements, we detected that the analysis of *pol*-derived sequences may offer at most a partial view of the patterns of evolution of these elements. We found that many Athila elements are characterized by either of two alternative structures, typical of non-autonomous retrotransposons: 1) LTRs plus a single ORF encoding Gag proteins, or, 2) LTRs plus two ORFs, encoding Gag and Env proteins. We also found that all potentially autonomous Athilas, those with *pol *sequences (including RTs), also have *gag *sequences, although they may or may not have *env *sequences.

These results led us to the idea of reassessing Athila evolution from the point of view of their *gag *sequences. We reasoned that *gag *sequences, common to all types of both complete and non-autonomous elements, would provide the most precise picture of the evolutionary history of Athila retrotransposons. We thus built phylogenetic trees based on Athila *gag *sequences. We must note here that in a previous study, based on RT sequences, Athila and the closest relative of Athila, the *env*-lacking retrotransposon that we named Little Athila [18] were confounded [20]. However, the recent addition of many novel sequences allowed us to confirm that Athila and Little Athila elements are not only often structurally different (Athilas often contain *env *sequences, while Little Athilas always lack *env*), but also possess very different sequences and thus are better defined as two different elements. Particularly, we found that they appear as two separate lineages not only in *Arabidopsis*, but also in species of the *Brassica *genus. This result demonstrates that Athila and Little Athila split at least 15–20 millions of years ago (our results are summarized in [22]). This result was also found by Zhang and Wessler [23] in their general comparison of the elements present in *Arabidopsis *and *Brassica*. Those authors also considered Athila and Little Athila as two different elements. Thus, all the subsequent results shown here refer solely to Athila elements, as defined by Marín and Lloréns [18] and Zhang and Wessler [23].

**Figure 1:**
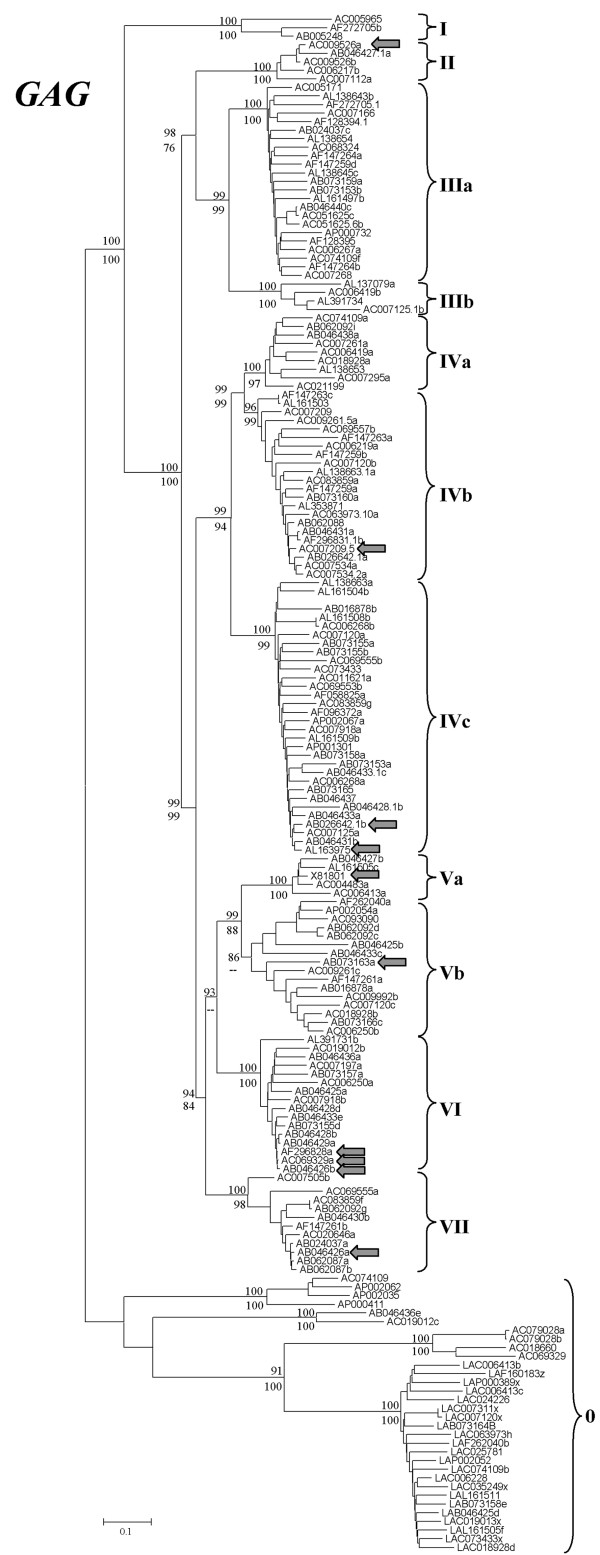
**Neighbor-joining phylogenetic tree based on the *gag *sequences of *Arabidopsis thaliana *Athila elements**. The names refer to the accession numbers from which the elements were obtained. Some times, letters have been added at the end to name different elements present in the same sequence. Numbers in the branches refer to bootstrap support (in percentages) for two different methods, neighbor-joining (NJ, top) and maximum parsimony (MP, bottom). The results of both methods were, in this case and the ones in the next two figures, almost identical, so they can be shown in a single tree. Arrows points to the ten elements without frameshifts or stop codons in their ORFs (discussed in the text).

Neighbor-joining and maximum parsimony phylogenetic trees based on *gag *sequences confirmed that Athila is a complex ensemble, formed by highly differentiated families. Twelve monophyletic, divergent Athila families became apparent in those trees (Figure 1; data for the elements can be found in [Additional file 1]). One of them ("Family 0" in Figure 1) included only highly defective copies and was not further analyzed. Table 1 contains the description of canonical copies for the other 11 families. Apart from the *gag *sequences of different families being very different, further demonstration for the high degree of diversification among Athila lineages was provided by the fact that LTRs of elements of different families were in general highly divergent and only partially alignable. We obtained estimates for the time when families diverged in the only two cases in which the full sequences of their LTRs could be compared (see Methods). Thus, families IVa and IVb were estimated to split about 2.7 millions of years ago, while families IIIa and IIIb diverged about 3.0 millions of years ago. These results agree well with the upper estimates for the time of insertion within a family, again according to LTR divergence, which reaches 2.4 millions of years (Table 1). If we now extrapolate from the data shown in Figure 1, assuming that divergence in *gag *sequences is roughly proportional to divergence time, an age of 6 to 10 millions of years for the most ancient splits among Athila families can be estimated. Thus, we can conclude that Athila families, as defined according to *gag *sequences, are ancient evolutionary lineages. An upper limit of less than 15–20 millions of years can be deduced from the fact that these eleven *Arabidopsis *families appear as a monophyletic group separated from all *Brassica *Athilas (data not shown).

**Table 1:** Canonical Athila retroelements. The numbers refer to the nucleotides of each sequence that correspond to Athila ORFs or LTRs.

		*Locations of the ORFs*					
			
**FAMILY**	*Acc. No*.	ORF1 (*gag*)	ORF2 (*pol*)	ORF2 (*pol*)	ORF3 (*env*)	Locations of the LTRs	Insertion range‡ (Myr)
			RT	Integrase			
**I**	AB005248.1	26036–27931	28901–29497	30719–31504	32343–33455	24125–25715/34256–35848	0.07 ± 0.03 – 1.60 ± 0.33
**II**	AC007112.6	13686–15547	X	X	17353–18318	11871–13507/20699–22334	0.33 ± 0.10 – 1.63 ± 0.20
**IIIa**	AC007268.5	54217–56048	X	X	58170–59432	52396–53654/61662–62920	0.80 ± 0.13 – 2.07 ± 0.33
**IIIb**	AC006419.4a	16642–18676	19664–20167	21481–22261	22764–24068	14874–16490/25936–27580	1.77 ± 0.20 – 2.07 ± 0.23
**IVa**	AC006419.4b	77199–79245	X	X	*	75761–77058/82966–84163	1.00 ± 0.13 – 2.40 ± 0.23
**IVb**	AC007209.6	20563–18531	17510–16914	15695–14953	13758–11905	22465–20708/10282–8571	0.17 ± 0.07 – 1.07 ± 0.13
**IVc**	AC007125.1	8267–10612	X	X	12350–14105	6319–8119/15919–17719	0.20 ± 0.10 – 1.63 ± 0.20
**Va**	X81801.1	1732–4539	X	X	5248–7332	1–1539/8954–10505	0.07 ± 0.03 – 1.73 ± 0.23
**Vb**	AF262040.1	39443–41436	X	X	*	38797–39257/45690–46143	0.80 ± 0.13 – 2.33 ± 0.33
**VI**	AC069329.6	42891–40537	X	X	38906–36963	44864–43063/35330–33522	0.20 ± 0.10 – 1.83 ± 0.23
**VII**	AB062087.1	46573–44021	43484–42995	41773–40991	*	48228–46745/38467–37031	0.17 ± 0.07 – 0.93 ± 0.13

*These elements contain a very short fragment of *env*, not long enough as to be included in the sequence alignments.

‡Range of insertion times detected within each family, estimated according to LTRs sequence similarity. In millions of years.

Most significantly, only four of the eleven *gag*-defined families (I, IIIb, IVb and VII) contained elements with *pol *sequences (see Table 1). These results suggest that most Athila elements, in fact complete families, are non-autonomous, and that they propagate by using the enzymatic machinery provided by elements of other families. Comparative analyses of LTRs demonstrated that non-autonomous families have been multiplying in the genome for periods of time of up to 2 millions of years (Table 1). Obviously, these results also show that all accounts of Athila element evolution published so far, based on RT sequences, offered a very incomplete view of the evolutionary dynamics of this complex ensemble of retroelements.

### Activity of Athila retrotransposons

If we assume that the available sequences correctly represent the diversity of the Athila elements present in *Arabidopsis thaliana*, we may infer the degree of activity of the different families of elements by their number of active copies. Significantly, most Athila sequences are non functional. Out of the almost 200 sequences of Athila elements analyzed, we detected only 10 potentially active elements, which contained ORFs without any frameshifts or stop codons. These elements also contain all characteristic conserved amino acids of Athila Gag proteins and, those that contain *pol *sequences, also contain the typical motifs of the active centers of reverse transcriptases and integrases. The 10 elements belonged to seven different families, as follows: 1 element from family II, 1 element from family IVb, 2 elements from family IVc, 1 element from family Va, 1 element from family Vb, 3 elements from family VI and 1 element from family VII codons (see arrows in Figure 1). Interestingly, no element in four of those families (II, IVc, Va and VI) has *pol *sequences, they just contain *gag *or *gag + env *sequences. Thus, only three copies among all Athila elements found so far are potentially autonomous, *pol*-containing copies. Two of them are from family IVb – corresponding to the "Athila4" element already characterized as potentially autonomous by Marín and Lloréns [18] and Wright and Voytas [20] – and family VII, respectively. These two families contain other elements with *pol *sequences, albeit defective. The third one belongs, according to its *gag *sequence, to family Vb, but, surprisingly, all but two elements in this family lack *pol *sequences. These peculiar elements, named Va-rec in Figure 1, will be discussed in detail in the next section.

LTR comparative analyses showed that the youngest elements in three of the families without potentially active copies, IIIa, IIIb and IVa, retrotransposed 0.8, 1.8 and 1.0 millions of years ago respectively (Table 1). This result may imply that these families are currently extinct. However, the presence of active Athila elements of these families in other *Arabidopsis *genomes cannot be excluded. For the fourth family without active copies (family I), a very recent insertion (estimated to have occurred 0.07 ± 0.03 millions of years ago; Table 1) was detected, suggesting that this family is still active. On the other hand, the most recent copies of the families with potentially active elements are in general quite young (average: 0.28 ± 0.09 millions of years) suggesting that most or perhaps all of them are still currently replicating.

The low number of potentially active copies and of recently inserted retrotransposons suggest that Athila elements, at least in the strains from which the examined sequences were derived, have in general a very low level of activity. We specifically searched for Athila cDNAs in order to obtain further evidence for the level of activity of these elements. A total of 169 ESTs corresponding to Athila cDNAs were found in the NCBI EST database and 121 of them could be unambiguously assigned to one of the defined Athila families (Table 2). Of them, just 48 ESTs could be assigned to *pol*-containing elements. However, none of them derived from the *pol *gene of those elements. Moreover, only 2 ESTs derived from one of the potentially active elements described above (the one in sequence AL163975; family IVc). If we consider that finding ESTs does not necessarily mean that these elements can transpose, that most of these ESTs derive from experimental conditions in which Athilas are known to be derepressed (see review by [24]) and that the NCBI database currently contains 1.3 millions of ESTs (an average of more than 40 per gene), it is clear that the level of Athila transcription and in general its ability to replicate must indeed be very limited.

**Table 2:** Results of selective regime analyses. "x", "y" and "z" refer to the three elements analyzed, with "z" being the one with coding potential, "y" a very close relative and "x" a more distant relative. In all cases except Va-rec, all elements in each analysis belong to the same family. For Va-rec, elements of the two families that give rise to the element were used. In this case, the *gag *sequences were not analyzed, due to the fact that they are of recombinant origin.

	**Sequences**			**Best model**	**ω in each branch**			
			
**Family**	**x**	**y**	**z**		**a**	**b**	**c**	**d**
II (*gag*)	AC007112a	AC006217b	AC009526a	M0	0.25	0.25	0.25	0.25
II (*env*)	AC007112.5	AC006217b	AC009526.4a	M0	0.20	0.20	0.20	0.20
IVb (*gag*)	AL161503	AC006219a	AC007209.5	M0	0.21	0.21	0.21	0.21
IVb (RT)	AL161503.2	AC006219	AC007209.5	M0	0.19	0.19	0.19	0.19
IVb (IN)	AL161503.2	AC069261.5a	AC007209.5	M0	0.09	0.09	0.09	0.09
IVb (*env*)	AL161503.2	AF147263.1b	AC007209.5	M0	0.24	0.24	0.24	0.24
IVc (*gag*)	AL138663.1b	AC069555b	AB026642.1b	M0	0.19	0.19	0.19	0.19
IVc (*env*)	AL138663a	AC069555b	AB026642.1b	M2	0.23	0.23	0.23	0.19
Va (*gag*)	AP002033.1	AL161505c	X81801	M0	0.38	0.38	0.38	0.38
Va (*env*)	AP002033.1	AL161505.2a	X81801.1	M0	0.30	0.30	0.30	0.30
Va-rec (*env*)	AB062087b (VII)	X81801 (Va)	AB073163a	M0	0.21	0.21	0.21	0.21
VI (*gag*)	AC007197a	AB046433e	AB046426b	M0	0.21	0.21	0.21	0.21
VI (*env*)	AC007197.5	AB046433.1a	AB046426.1a	M0	0.21	0.21	0.21	0.21
VII (*gag*)	AC069555a	AB046430b	AB046426a	M0	0.31	0.31	0.31	0.31
VII (RT)	AC069555a	AB046430b	AB046426a	M2	0.46	0.46	0.46	0.00

### Recombination among elements of distantly related families

**Figure 2:**
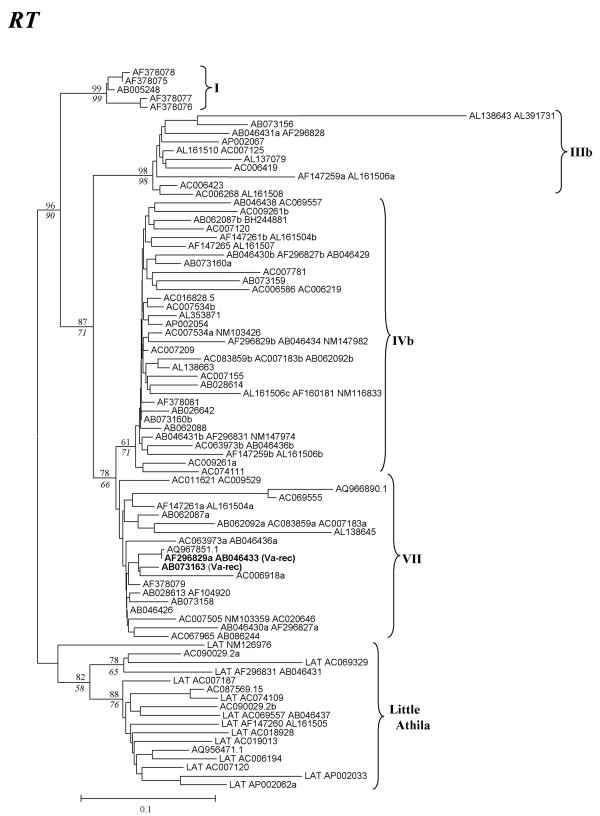
**Neighbor-joining tree obtained for RT sequences of Athila elements in *Arabidopsis thaliana***. Names and bootstrap values (NJ/MP) as in Figure 1.

**Figure 3:**
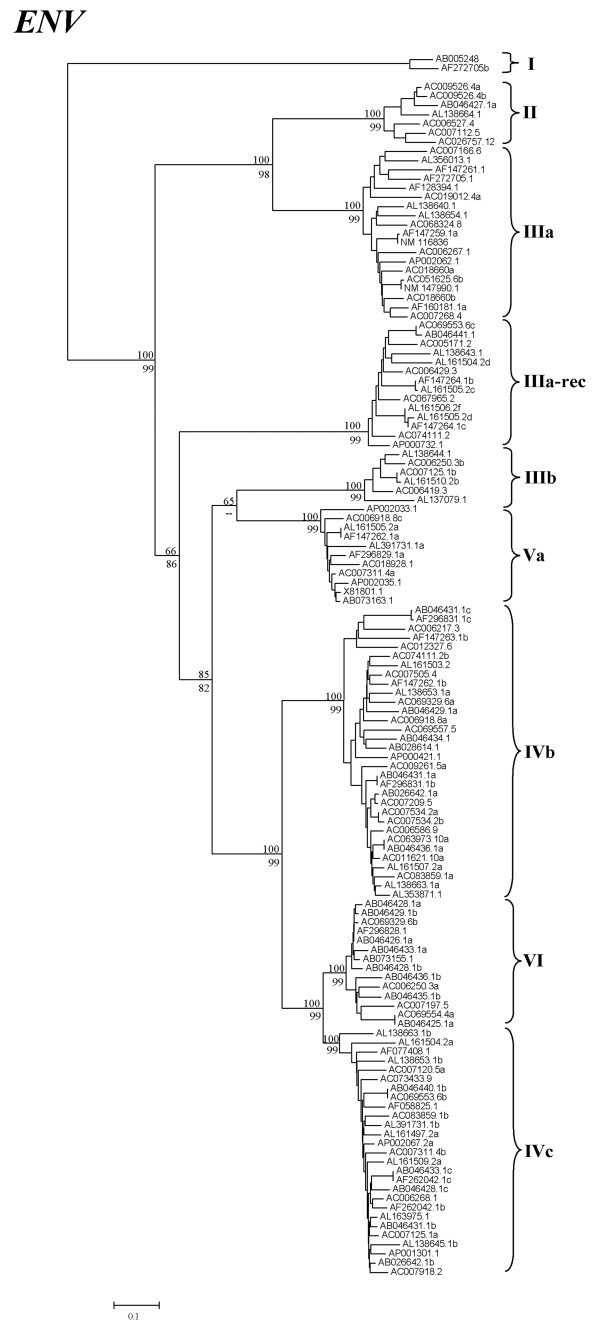
**Neighbor-joining tree based on *env *sequences of *Arabidopsis thaliana *Athilas**. Names and bootstrap values again NJ/MP as in Figure 1.

As just detailed, one of the potentially active elements (Va-rec) contained a *gag *sequence that was included in family Vb in our phylogenetic tree (Acc. No. AB073163; Figure 1). However, closer inspection of this particular copy demonstrated that it was not a typical Vb element. We found that, while no element in family Vb has *env *sequences (Table 1), the Va-rec element contained an *env *sequence very similar to those found in family Va. In addition and as already indicated above, Va-rec and another very similar but defective element (Acc. No. AB046433; Figure 1), were the only two elements having Vb-like *gag *sequences but containing also *pol *sequences. We noticed that these *pol *sequences were actually very similar to those found in family VII elements. These strange results, suggestive of a recombination process, led us to consider in more detail the relationships among *gag*, *pol *and *env *sequences for the whole set of Athila retrotransposons (see Figures 2, 3 and [Additional files 2, 3] for the details of the RT and *env *sequences).

**Figure 4:**
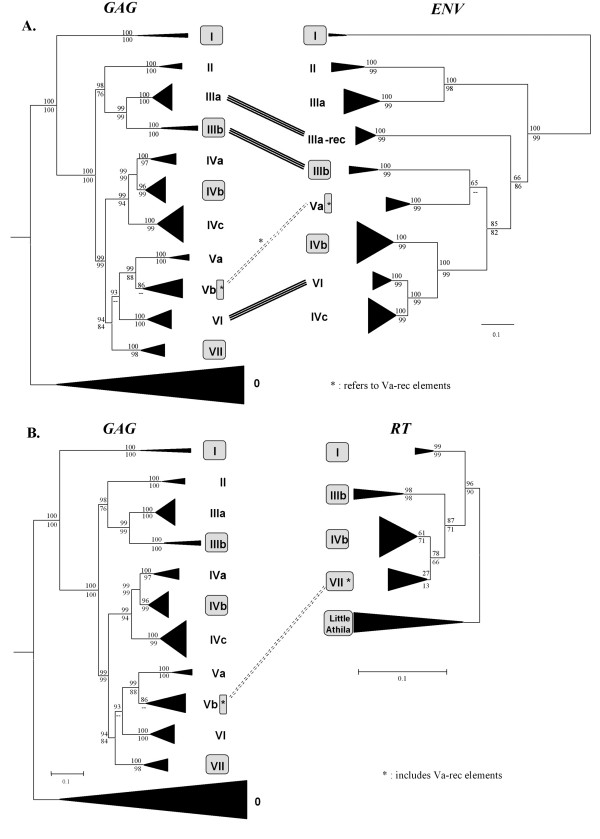
**Comparisons of the phylogenetic trees obtained using different Athila coding sequences**. A) Comparison of the Gag and Env trees. Lines connecting both trees indicate different relative positions of families or elements, corresponding to recombination events. The dashed lines refer to the recombination event that generated the Va-rec elements. Bootstrap support for the topology is shown in percentages (Top: neighbor-joining; Bottom: maximum parsimony). In gray boxes, we show the families that contain *pol *sequences. B) Comparison of the Gag and RT trees.

**Figure 5:**
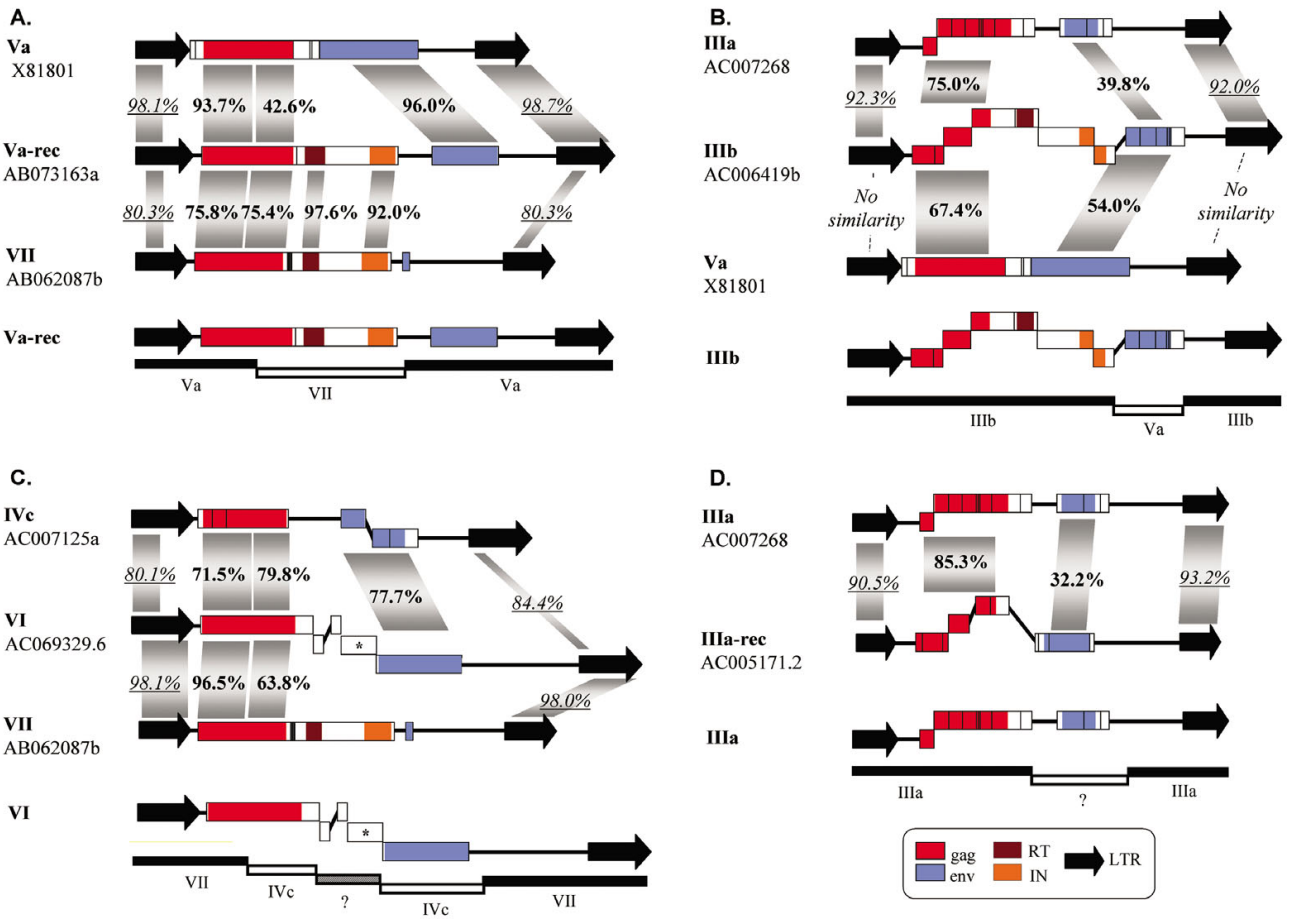
**Recombination events**. For panels A to D, we show on the top the similarity between a canonical member of each recombinant lineage and the canonical members of the parental lineages that participated in the recombination event. On the bottom, we summarize the most likely origin of the different parts of the recombinant elements. Numbers in bold: percentage of amino acidic similarity. Numbers in italics and underlined: percentage of nucleotide identity. See details in the text.

When trees generated with the *gag *(Figure 1), RT (*pol*-derived; Figure 2 and see [Additional file 2] for details) and *env *sequences (Figure 3 and [Additional file 3]) were compared, it was found that they were often congruent (Figures 4A, 4B). However, we observed several significant differences (outlined also in Figures 4A and 4B). They could only be explained by four independent recombination events among elements of distant families. Comparisons among LTRs and coding sequences allowed us to determine the evolutionary histories for those events:

1) Origin of Va-rec elements: We were able to determine that although these elements appeared in the *gag*-based phylogenetic trees as members of the Vb family, this was an artifact caused by them having a *gag *sequence of mixed origin. They emerged by the acquisition by an element of family Va of part of the *gag *gene and a complete *pol *gene derived from a family VII element (Figure 5A). Va-rec is therefore an element of recombinant origin, generated from a non-autonomous progenitor of the Va family that lacked *pol*. As we already mentioned, a second Va-rec element, but with stop codons and frameshifts was also detected. This finding demonstrates that Va-rec elements have been active after the recombination process that originated them. The only apparently active Va-rec element found has a relatively recent origin, becoming inserted 0.27 ± 0.07 millions of years ago.

2) Acquisition by a family IIIb element of *env *sequences originated from a family Va element. This event explains the shift in the position of family IIIb elements in the *gag*- and *env*-based trees (Figures 4A, 5B).

3) Recombination between elements of the IVc and VII families, to give rise to family VI. Family VI elements have LTRs and part of the *gag *sequences that are extremely similar to family VII elements, while the rest of the *gag *and the *env *sequences are very similar to those in family IVc (Figure 5C)

4) Acquisition of some family IIIa elements of an *env *of uncertain origin, generating an additional branch of elements in the *env*-based tree, that we named IIIa-rec (Figure 5D).

In summary, these results demonstrate that recombination between elements of different families has occurred frequently in the past: at least 4 of the 13 lineages observed in this study (i. e. the 11 families described in Table 1 plus the IIIa-rec and Va-rec lineages, which cannot be detected in *gag*-based trees), are of recombinant origin. This is probably an understimate, because ancient recombination events or those involving short sequences would remain undetected with our methods. In any case, recombination has been so frequent that none of the phylogenetic trees obtained properly reflected the diversity of Athila retrotransposons. Only tree comparisons allowed us to understand the evolution of these elements.

### Selective pressures acting on Athila retroelements

The fact that 70% of the potentially active copies encode for Gag or Env proteins but not for Pol proteins raises the question of whether the *pol*-less elements are simply parasites of the *pol*-containing copies or, alternatively, they may be contributing to their own propagation or to the propagation of other Athila elements. This contribution would require the production of active Gag or Env proteins by the non-autonomous elements. Of course, to conclude that these elements may contribute functional proteins is not enough to find out that the non-autonomous copies contain potentially coding ORFs or finding ESTs derived from these elements. Even then, all they could be propagating strictly in a parasitic way, i. e. depending solely on proteins provided in *trans *by other elements with their own genes being non-functional or fully repressed.

The question of whether particular elements contain functionally relevant sequences can be tackled by considering the selective pressures acting on them. Particularly, if they are functionally irrelevant, we would expect the coding regions of non-autonomous copies to evolve at a neutral rate. If, on the other hand, we find that evolution in certain coding regions of non-autonomous copies are under negative, purifying selection, then this would be a strong evidence for them being functionally significant. However, a problem that arises in this type of studies is the potential confusion caused by the past effects of negative selection. Imagine that, after an element replicates, there follows a period of time in which the coding sequences of both copies remain active. In that period, selection on their coding regions would generate an accumulation of changes in permissive positions (e. g. third positions of codons) respect to more constrained positions. If later one of these copies becomes inactive, and starts evolving neutrally, changes will accumulate at random. However, if, after some time, we compare both sequences, we may still find evidence for negative selection, due to the imprint of pass negative selection being still detectable. To avoid this problem, we followed the strategy of comparing the ORFs of potentially active Athila elements only with copies that were very closely related. More precisely, we analyzed trios of sequences, consisting on the sequence that we want to analyze, one of its closest relatives and a third, more distant relative. With these trios, for which phylogenetic relationships are obvious, we performed codon-based analyses of the relevant ORFs to establish the specific rates of non-synonymous vs. synonymous changes (ω) for the different branches (see details in Methods). All the relevant results are shown in Table 3 and the whole set of analyses is detailed in [Additional file 4]. The summary of these results is very simple: in all cases, the best model implies strong negative selection on the branch that corresponds to the elements with potentially active ORFs (branch "z" in the schematic tree shown at the top of Table 3). These results strongly reinforce the idea that these ORFs indeed encode functional proteins, which may contribute to either the replication of the copies that carry them or of other elements in *trans*.

**Table 3:** Summary of ESTs derived from Athila elements

***Family***	***Most similar genomic DNA***	***ESTs***
I	AF272705b (env)	EG477448, EG477439, EG477443, EG477438, EG477426
I	AB005248 (env)	EG477445
IIIa	AC007166.6 (env)	EG526171, EG526175, EG526169, EG526179, EG526177, EG526176, EG526150
IIIb	AC007125.1b (env)	EG462911, EG455888, EG462908
IIIb	AL391734 (env)	EG462905, EG462904, EG455886
IIIb	AL137079.1 (env)	EG463619, EG463628, EG463610, EG484314, EG484306, EG484300, EG461948, EG461965, EG461956, EG484310, EG463626, EG484303, EG461951, EG484304, EG484316, EG484314, EG484313, EG484299, EG484318, EG463627, EG463617, EG461953, EG459865, EG459891, EG461968, EG461958, EG461955, EG461949, EG463621, EG463625, EG463616, EG461967, EG484315, EG484298, EG459890, EG463611
IVc	AB046427.1a (gag)	EG452894, EG452887, EG452883, EG452874, EG452872, EG452892, EG452885, EG452879, EG452900, EG452890, EG452873, EG452871
IVc	AB046428.1b (gag)	BE526916
IVc	AP002067a (gag)	BP837984, BP837278
Va	AL161505.2a (env)	EG458656, EG458650
Va	AC004483a (gag)	EG491254, EG491238, EG491236, EG491239, EG491247, EG491249, EG491252, EG491244, EG491246, EG491253, EG491242
VI	AB073157a (gag)	EG447146, EG446192, EG448096, EG418344
VI	AB073166.1 (env)	EG526158, EG526152
VI	AB046428.1b (env)	EG526117, EG526154, EG526119
VI	AB046425.1 (env)	EG479658, EG504857, EG479617, EG504777, EG479662, EG479648, EG479652, EG479650, EG479663, EG479611, EG479654, EG479607, EG504604, EG504604, EG479605, EG479657, EG504582, EG479655, EG479604, EG479649, EG479646, EG479644, EG479661, EG504856, EG479643, EG479651, EG479608
*unassigned*	-	EG472931, EG472931, EG459693, EG459691, BP823996, BP822138, BP819107, BP826056, EG491235, EG491241, EG423786, EG491243, EG491248, EG491250, EG491245, EG491237, EG459228, EG459225, EG479658, EG504857, EG479617, EG504777, EG479662, EG479648, EG479652, EG479650, EG479663, EG479611, EG479654, EG479607, EG504604, EG479605, EG479657, EG504582, EG479655, EG479604, EG479649, EG479646, EG479644, EG479661, EG504856, EG479643, EG479651, EG479608, EG459688, EG459694, EG459692, EG459690

## Discussion

We may now recapitulate the observations described in the previous chapter. First, we have shown that Athila is composed by at least 11 different families, defined as monophyletic groups of closely related elements (Figure 1). Most of these families emerged in the distant past. We dated the splits between families as having occurred at least 2.7 millions of years ago. Even considering all types of elements, autonomous or not, Athilas are not present in large numbers. Our results agree very well with a previous estimation of about 200 structurally intact copies of Athila per genome [25]. There are no predominant families, so the number of elements for each family is low, ranging from 3 to 31 in our dataset and with an average of 13.2 ± 2.8 (see also Figure 1). Finally, there are only 3 potentially autonomous, active copies in the whole dataset. All these results, together with the low number of ESTs found, suggest that Athila activity is very low. If our results can be extrapolated to other *Arabidopsis *genomes, we can conclude that Athila as a whole is not a particularly succesful parasite, i. e. it survives at low numbers, and that individual Athila families are at the verge of extinction, at least in individual genomes (although perhaps they are doing fine in the whole species).

Our second main result is that we have shown that families that contain very similar elements without *pol *sequences have been replicating in the *Arabidopsis *genome for more than 2 millions of years. These elements are characterized by containing *gag *or *gag *+ *env *sequences and several copies have kept apparently intact ORFs with coding potential and which are under a purifying selection regime. Thus, the proteins derived from elements of these families may be contributing to its own replication or to the replication of other Athila elements. Finally, the third main result is that recombination between elements of distantly related families is relatively frequent.

These results are quite different from those observed for most other LTR retrotransposons. For example, in the thoroughly analyzed *Saccharomyces cerevisiae*, *Caenorhabditis elegans *or *Drosophila melanogaster *genomes, most LTR retrotransposons are active, and there are no descriptions of abundant non-autonomous copies with coding potential [9,10,26]. Recombination between elements of different families of LTR retrotransposons (Ty1 and Ty2) leading to the generation of a new lineage (Ty1/Ty2) was first observed in *S. cerevisiae *[27,28]. However, differently for what we have found for recombinant Athilas, all Ty1/Ty2 recombinant copies are recent [29] so their long-term evolutionary potential is unclear. Similar cases, in which new lineages of active elements are produced by recombination, have been described in other species [30,31]. Finally, a case in which a novel non-autonomous element that retains coding potential has emerged by recombination has been described in *Hordeum *[32,33], but, again, this element is very young and therefore their ability to propagate for long periods of time is unknown. Significantly, recombination leading to ORFs encoding for "hybrid" proteins of mixed origin, as occurred in Athila families Va-rec and VI (Figures 5A, 5C) was not found in any of these cases.

We may now ask what are the evolutionary processes that explains the particular pattern of evolution observed for Athila elements. First, we may consider whether our results are compatible with the hypothesis of full evolutionary independence of Athila families. To consider fully independent those families for which we have found only non-autonomous copies, we ought to hypothesize that hitherto undiscovered autonomous copies exist for those lineages. This is formally possible but very unlikely. These copies should be promoting the expansion of highly similar, structurally identical defective copies for periods of millions of years while not leaving any detectable *pol*-containing remnant in the genome. The best argument against this happening is that such peculiar pattern is never observed for the families that do have *pol *sequences. That is, although many copies in families with *pol*-containing elements are defective – having accumulated stop codons and frameshifts –, we never observed *pol*-less elements within those families. We may thus reason that, if in families for which *pol*-containing elements are known, we never detect a set of related *pol*-less elements, it is highly unlikely that precisely in those families for which we have not detected *pol*-containing elements, they actually exist. Therefore, the simplest explanation for the observed pattern is that *pol*-less elements are mobilized, at least in part, in *trans*, by enzymes provided by elements that belong to different, *pol*-containing, families.

We may then ask whether this is just another case of parasitism in which non-autonomous copies use the enzymatic machinery of the active ones without providing any compensation or, alternatively, some kind of cooperation between autonomous and non-autonomous elements might exist. There are two ways in which such cooperation may arise. First, non-autonomous elements with coding potential could contribute to the replication of autonomous copies. To demonstrate this process would require direct biochemical analyses, which is beyond the scope of this work. Our data show however that two necessary conditions for the process to occur are present: 1) there are non-autonomous elements with coding potential, with proteins which are under negative selective pressures; and, 2) the products of distant Athilas are biochemically compatible, as it is demonstrated by the emergence, by recombination, of new families with genes of different origin.

The second way in which cooperation might arise is indirect: generation of coding, non-autonomous copies could be advantageous for the long-term survival of Athila elements as a whole, if the non-autonomous copies occassionally contribute to the generation of novel successful families. Our results demonstrate that this type of event has occurred. We have shown that Athila autonomous and non-autonomous families are linked by recombination events and that several successful recombinant Athila lineages, defined as lineages able to replicate and survive for long periods of time, have arisen. They are of three different types: 1) novel autonomous lineages such as the Va-rec elements; 2) non-autonomous recombinant lineages that have survived while one or perhaps both progenitor families have become extinct, as seems to be the case for the family that provided the *env *sequences now found in IIIa-rec elements (Figure 5D); or, 3) simply recombinant non-autonomous lineages that are able to propagate in the genome as efficiently as autonomous ones (e. g. family VI, which has been replicating for at least two millions of years). Among all these results, it is most interesting that we may have detected the birth of a new evolutionary entity: if *env *sequences indeed provide Athila elements with the possibility of becoming infective, Va-rec elements would be an example of how recombination between an autonomous retrotransposon (*env*-less, from family VII) and a non-autonomous element (*pol*-less, from family Va) generates a novel active retrovirus (with *gag*, *pol *and *env*; Figure 5A). In any case, these events demonstrate that non-autonomous copies are not strictly parasitic. They are contributing to the long-term survival of Athila elements in *Arabidopsis*.

## Conclusion

In summary, our results suggest that distant Athila families may be cooperating to survive in the *Arabidopsis *genome. Cooperation among other type of mobile elements, bacterial IS elements, has recently received attention, with the conclusion that it may appear under precise selective regimes [34]. Recent models also suggest situations in which mutualism may occur [35]. In fact, we think that the accepted view that all elements behave strictly selfishly may be due to the great difficulties involved in discovering patterns of sequence evolution compatible with cooperative processes. It is possible that other mobile elements follow dynamics similar to the one we have just described. For example, evidence for a related pattern of interchange to generate novel lineages is also available for human endogenous retroviruses ([36,37]; see also discussion in [38]). Therefore, this may be the first formal description of a widely-used survival strategy for eukaryotic mobile elements.

## Methods

### Data mining and phylogenetic analyses

We built databases of Gag, reverse transcriptase and Env proteins using BlastP and TblastN searches against the databases available at the National Center for Biotechnology Information (NCBI). We used as queries multiple representative Athila elements until the searches become saturated. After each search, we aligned the sequences obtained and removed duplicates and partial sequences. All alignments were performed with ClustalX 1.83 [39] using default parameters. Alignments were manually corrected when necessary with GeneDoc 2.6 [40]. We used two methods of phylogenetic inference, neighbor-joining and maximum parsimony, implemented in MEGA2 [41] following the methods described in [42]. For both methods, statistical support for the branches was assessed performing 1000 bootstrap replicates.

### Structural analyses

To determine the structure of Athila elements, we first used Blast2sequences searches [43], comparing each element with known *gag*, RT, integrase and *env *Athila sequences. ORFs were detected with ORF finder [44]. LTR locations were determined by looking for similarity within an element, also with Blast2sequences.

### Estimation of the insertion time or divergence time between elements

We estimated the insertion time for an element or the divergence time for elements of two different families following the strategy described by San Miguel *et al*. [45]. The nucleotide sequences of either both LTRs of each element or single LTRs of two different elements were aligned and the Kimura two-parameter distance [46] was estimated using MEGA2. The distance obtained was then divided by two (because it refers to changes accumulated in both LTRs) and then again divided by the substitution rate at synonymous sites estimated for the brassicaceae *Chs *and *Adh *genes [47], that is, 1.5 10^-8 ^per site per year.

### Characterization of recombination events

Recombination events were deduced from incongruent phylogenetic positions of two proteins of a same element or group of elements [48]. We searched for the recombination breakpoints by analyzing pairwise alignments of amino acidic sequences and also, at the nucleotide level, following a sliding-window approach implemented in SimPlot [49], which utilizes the DNAPARS and NEIGHBOR programs of the Phylip package [50].

### Estimations fo synonymous and nonsynonymous nucleotide substitutions

The rate of synonymous and nonsynonymous substitution were estimated using the PAML3.1 package [51], following the strategy of branch-dependent analyses described previously in [52]. Three models were analyzed. M0 refers to a model in which all branches are assumed to have the same rate. M1 is a model in which all branches are assumed to evolve at different rates. Finally, M2 is a model in which the branch that leads to the active element ("z" in Table 2) is assumed to evolve at a different rate than the rest.

## Authors' contributions

AM performed all the analyses presented here and contributed to the text. IM devised and supervised the research and wrote the manuscript.

## Supplementary Material

Additional file 1 Supplementary_table_1 Click here for file

Additional file 2 Supplementary_table_2 Click here for file

Additional file 3 Supplementary_table_3 Click here for file

Additional file 4 Supplementary_table_4 Click here for file
