# Supplementary material for: How Athila retrotransposons survive in the Arabidopsis genome
Source: BMC Genomics. 2008 May 14;9:219. doi: 10.1186/1471-2164-9-219 (PMC2410132; doi:10.1186/1471-2164-9-219)
Supplement: Additional file 1 — Supplementary_table_1 [file 1471-2164-9-219-S1.pdf]

Supplementary Table 1. Positions of the elements shown in Figure 1 (Gag tree) in each one of the clones analyzed. The letters a, b, c,… in the Accession Numbers refer to cases in which there is more than one element in the same sequence. In this and the following tables, we have used these abbreviations: **: proteins not present; ?: not found (e. g. not sequenced yet); n. d.: not determined. In parenthesis: very short fragments.

	Accession Number (Acc. No.)	*gag*	RT	*env*
I	AC005965	72489-70631	**	**
	AF272705b	12278-10350	9381-8818	5937-4771
	AB005248	26036-27931	28901-29497	32343-33455
II	AC009526a	86224-84386	**	82508-81348
	AC009526b	77139-75263	**	73352-72225
	AB046427.1	45895-44414	**	43186-42130
	AC006217b	91363-93240	**	95124-95951
	AC007112a	13686-15547	**	17353-18318
III-a	AC007268	54217-56048	**	58170-59432
	AF147264b	15757-13977	**	11941-10234
	AC074109f	53674-52105	**	49759-48503
	AF272705.1	127802-125975	**	123689-122644
	AF128394.1	13038-11295	**	8982-7963
	AC051625.6b	28107-29864	**	32184-33233
	AF128395	6684-8508	**	**
	AC006267a	53840-55627	**	57723-58985
	AP000732	43498-45226	**	**
	AC068324	40206-38397	**	36512-35106
	AF147264a	17231-19060	**	20777-22649
	AL161497b	157820-145065	**	**
	AB024037c	33826-35649	**	37758-38492
	AB073153b	6724-4913	**	2805-1577
	AB073159a	3723-1900	?	?
	AF147259d	3065-4915	**	7085-8211
	AL138645c	34386-36203	**	**
	AC051625c	37261-39085	**	**
	AB046440c	6879-5918	**	?
	AC005171	81566-79822	**	77737-76208
	AL138654	39955-41781	**	44959-45088
	AL138643b	84340-82558	**	80914-79448
	AC007166	3111-4935	**	6992-8318
III-b	AL137079a	39425-41461	42465-43052	45690-47261
	AC007125.1b	102259-100832	99805-99209	96343-95278
	AC006419b	16642-18676	19664-20167	22761-23752
	AL391734	24712-22681	21691-21470	18776-17061
IV-a	AC074109a	5993-4086	?	?
	AB062092i	15934-13734	**	(12432-12229)
	AB046438a	5774-3693	**	(2362-2189)

	AC007261a	18341-20402	**	(21731-21904)
	AC006419a	77199-79245	**	(80583-80762)
	AC018928a	5595-7688	**	(9013-9192)
	AL138653	3024-5020	**	(5660-5794)
	AC007295a	13885-11923	**	(10585-10457)
	AC021199	3257-1268	**	?
IV-b	AF147263c	65668-63834	**	63069-61252
	AL161503	170201-172035	**	172800-174617
	AC007209	20563-18531	17510-16914	13758-11905
	AF147259b	26035-24145	22996-22400	**
	AC009261.5a	17563-19424	20022-20618	23791-24861
	AL138663.1a	47920-50003	51025-51621	54892-55962
	AC063973.10a	46355-44433	43424-42828	39615-38545
	AF296831.1b	50961-48907	47887-47291	43966-42896
	AC007209.5	35217-37249	38270-38866	42154-43224
	AB026642.1a	15564-13491	12470-11874	8594-7524
	AC007534.2a	90480-92545	93566-94162	97466-98536
	AC069557b	33315-35251	36270-36866	40108-41979
	AF147263a	18674-16713	15468-15013	10956-9092
	AC006219a	74716-76721	77740-78336	(79198-79446)
	AC007120b	40917-38878	36249-35655	30972-29978
	AB073160a	26697-28778	29773-30369	33500-35352
	AF147259a	11347-13426	**	(112840-113223)
	AC083859a	30298-32331	33444-33947	37150-39012
	AL353871	87448-89481	90516-91097	94151-96002
	AC007534a	102696-104762	105818-106375	109546-111390
	AB046431a	80931-78877	77854-77261	74068-72215
	AB062088	93863-95896	96917-97513	100710-102563
IV-c	AL161504b	35755-37717	**	39889-40542
	AL138663a	28613-30553	**	32692-34610
	AB016878b	75076-73129	**	70874-69076
	AL161508b	108361-110347	**	123926-125841
	AB046427.1b	47749-49308	**	51762-52807
	AB026642.1b	45012-43016	**	40647-39577
	AB046428.1b	19339-17342	**	15121-14051
	AB046433.1c	14137-12713	**	10383-9312
	AC006268b	85184-98503	**	100565-102480
	AC007120a	26974-24994	**	22942-20999
	AB073155b	43524-41529	**	**
	AB073155a	83422-81460	**	79292-78800
	AC069555b	983-2971	**	5106-6934
	AC073433	108637-110607	**	112660-114574
	AF096372a	17019-15024	**	13290-11377
	AC011621a	47916-49913	**	51896-54072
	AC069553b	13702-15728	**	17746-19665
	AF058825a	61838-63817	**	65978-67845
	AC083859g	80988-78993	**	77397-75932
	AP002067a	6730-4722	**	2674-1022

	AC007918a	64256-62265	**	?
	AL161509b	59403-57405	**	55422-53506
	AB073153a	20535-18564	**	16952-15066
	AP001301	60121-62118	**	64142-66001
	AB073158a	49744-47751	**	45834-43933
	AC006268a	96509-98506	**	100565-102480
	AB046437	7687-9683	**	(10983-11186)
	AB073165	8552-6637	**	4590-2674
	AB046433a	43851-41890	**	39801-37885
	AC007125a	8267-10264	**	12350-14105
	AB046431b	47835-49809	**	52038-53909
	AL163975	10464-8470	**	6492-4576
V-a	AL161505c	153648-155629	**	157159-159240
	AB046427b	26174-24189	**	22658-18625
	X81801	1732-4539	**	5248-7332
	AC004483a	28388-26403	**	**
	AC006413a	33403-35349	**	36965-38658
V-b	AP002054a	80580-82574	**	**
	AF262040a	39443-41436	**	(42645-42770)
	AC093090	2756-4718	**	**
	AB062092d	28011-29999	**	(31167-31295)
	AB062092c	21071-23059	**	(24227-24355)
	AB046425b	12492-14485	**	(16353-16529)
	AB046433c **Va-rec**	59651-61654	67575-68162	71242-73107
	AB073163a **Va-rec**	10838-8862	7862-7266	4235-2370
	AC009261c	40238-42094	**	(43657-43992)
	AF147261a	78010-79859	**	**
	AB016878a	83349-85214	**	**
	AC009992b	51900-53884	**	**
	AC007120c	15959-13992	**	(12565-12225)
	AC018928b	106847-104856	**	**
	AB073166c	41232-39296	**	**
	AC006250b	35011-36872	**	(38343-38674)
VI	AL391731b	2004-36	?	?
	AC019012b	21720-19750	**	17671-15913
	AB046436a	82791-84773	**	86770-93085
	AB073157a	19901-17991	**	11207-9834
	AC006250a	50692-48737	**	47185-45258
	AC007197a	59373-61355	**	63450-65391
	AB046425a	21093-23075	**	25010-26920
	AC007918b	44370-42389	**	**
	AB046428d	32677-30697	**	28695-26752
	AB046433e	54716-52767	**	50598-48655
	AB073155d	6994-8995	**	11085-13028
	AB046428b	45802-47785	**	49849-51792
	AB046429a	1036-2985	**	5080-7023
	AF296828a	11835-13817	**	15820-17763
	AC069329a	42891-40909	**	38906-36963

	AB046426b	58164-56183	**	54180-52238
VII	AC007505b	89798-131490	**	**
	AC069555a	71676-69681	68681-68085	**
	AC083859f	44084-42090	**	**
	AB062092g	70994-69000	**	**
	AB046430b	10740-12673	13684-14253	(16626-16808)
	AF147261b	38122-36130	35131-34535	(32151-31969)
	AC020646a	21082-19108	18110-17514	(15132-14952)
	AB024037a	2797-803	?	?
	AB046426a	84315-82321	81321-80725	(78341-78159)
	AB062087a	36859-34865	**	**
	AB062087b	46573-44579	43489-42995	(40611-40429)
0	AP000411	37369-39498	**	**
	AP002035	52696-54626	**	**
	AC074109	111193-109267	**	**
	AP002062	25744-27594	**	**
	AB046436e	56192-53489	**	**
	AC019012c	27100-25453	**	**
	AC079028a	34923-33187	**	**
	AC079028b	28865-27129	**	**
	AC018660	65160-63341	**	**
	AC069329	7482-9245	**	**
	LAP000389x	5561-3357	**	**
	LAC006413c	66030-63887	**	**
	LAC006413b	60389-62613	**	**
	LAF160183z	61225-59179	**	**
	LAC024226	32793-34984	**	**
	LAC063973h	18889-16637	**	**
	LAC007311x	26664-24492	**	**
	LAC007120x	100213-102331	**	**
	LAF262040b	33522-35747	**	**
	LAB073164b	65572-63315	**	**
	LAC025781	58346-60623	**	**
	LAB073158e	73048-75376	**	**
	LAC074109b	100705-103370	**	**
	LAP002052	27780-25612	**	**
	LAC006228	81306-79141	**	**
	LAC035249x	68285-70550	**	**
	LAC073433x	80786-83043	**	**
	LAC018928d	86298-88551	**	**
	LAC019013x	78073-75819	**	**
	LAL161511	175500-173242	**	**
	LAL161505f	73659-71374	**	**
	LAB046425d	43626-45881	**	**
