# Supplementary material for: How Athila retrotransposons survive in the Arabidopsis genome
Source: BMC Genomics. 2008 May 14;9:219. doi: 10.1186/1471-2164-9-219 (PMC2410132; doi:10.1186/1471-2164-9-219)
Supplement: Additional file 2 — Supplementary_table_2 [file 1471-2164-9-219-S2.pdf]

Supplementary Table 2. Positions of the elements shown in Figure 2 (RT tree) in each one of the clones analyzed

	Acc. No.	*gag*	RT	*env*
**I**	AF378076	?	1-762	?
	AF378077	?	1-762	?
	AF378075	?	1-762	?
	AF378078	?	1-762	?
	AB005248	26036-27931	28901-29497	32343-33455
**III-b**	AF147259a	36525-37698	38293-38889	**
	AL161506a	n.d.	173375-173971	**
	AC006268	85175-87217	91998-92594	**
	AL161508	119870-122605	115359-115955	**
	AB073156	77449-79194	80469-81065	83785-85552
	AL138643	97365-95632	95313-94726	(92013-91879)
	AL391731	9286-7553	7234-6647	(3934-3800)
	AB046431a	20528-22644	23312-23875	(26359-26571)
	AF296828	56467-58583	59251-59814	(62298-62510)
	AC006423	n.d.	n.d.	n.d.
	AL161510	21175-19250	18616-18020	15319-13576
	AC007125	n.d.	99805-99209	n.d.
	AC006419	16642-19069	19664-20167	22761-23752
	AP002067	n.d.	n.d.	n.d.
	AL137079	39425-41461	42465-43052	45690-47261
**VII**	AC011621	56777-57608	58215-58811	(61096-61380)
	AC009529	41418-42249	42856-43452	(45737-46021)
	AB062092a	**	54972-54379	(52074-51790)
	AC083859a	**	28079-27486	(25181-24897)
	AC007183a	**	100254-99661	(97356-97072)
	AL138645	75239-74193	73397-72801	**
	AB062087a	46573-44186	43507-42995	(40641-40429)
	AF147261a	38112-35737	35131-34535	(32181-31969)
	AL161504a		161636-162232	n.d.
	AC069555	71676-69288	68681-68085	**
	AQ966890	?	n.d.	?
	AC063973a	10126-102195	102802-103398	**
	AB046436a	88244-89316	89923-90519	(92732-93085)
	AF378079	?	n.d.	?
	AB028613	**	35116-34520	(32166-31954)
	AF104920	10859-8472	7865-7269	(4915-4703)
	AB073158	52232-54181	55182-55778	(58005-58286)
	AB046426	84315-81928	81321-80725	(78371-78159)
	AC007505	132917-131097	130492-129896	(127514-127332)
	NM103359	?	n.d.	?
	AC020646	n.d.	18110-17514	n.d.
	AB046430a	11120-13066	13684-14253	(16602-16808)
	AF296827a	79414-81360	81978-82547	(84896-85102)
	AC067965	8825-11203	11818-12414	(14764-14946)

	AB086244	42517-40139	39524-38928	(36578-36396)
**Va-rec**	AF296829a	6547-7461	7765-8361	11431-13296
	AB046433	66357-67271	67575-68162	71242-73107
	AC006918a	n.d.	73801-72051	n.d.
	AQ967851	?	n.d.	?
	AB073163	10838-8196	7862-7266	4235-2370
**IV-b**	AB046438	58998-56769	56140-55544	52302-50431
	AC069557	n.d.	36270-36866	n.d.
	AC009261b	64317-65980	67151-67747	n.d.
	AB062087b	70497-68672	68368-67772	64631-62771
	BH224881	n.d.	n.d.	n.d.
	AC007120	40917-38485	36249-35764	31073-29978
	AF147261b	n.d.	45866-46462	(49652-50020)
	AL161504b	n.d.	150901-150305	n.d.
	AF147265	27515-25513	25161-24565	n.d.
	AL161507	n.d.	91059-90463	n.d.
	AB046430b	5500-4694	4067-3531	1053-1
	AF296827b	n.d.	72361-71825	n.d.
	AB046429	n.d.	94615-94079	n.d.
	AB073160a	26697-29024	29773-30369	33500-35352
	AC007781	n.d.	18890-19453	n.d.
	AB073159	51155-48947	48318-47722	n.d.
	AC006586	2317-4714	5341-5937	(6799-7047)
	AC006219	n.d.	77740-78336	n.d.
	AC016828.5	n.d.	n.d.	n.d.
	AC007534b	102696-105479	105818-106375	109546-111390
	AL353871	n.d.	n.d.	n.d.
	AP002054	n.d.	23264-22671	19417-18449
	AC007534a	90480-93262	93566-94162	97334-99187
	NM103426	n.d.	n.d.	n.d.
	AF296829b	n.d.	77592-76996	72909-71283
	AB046434	n.d.	22852-22256	n.d.
	NM147982	n.d.	n.d.	n.d.
	AC007209	20563-17814	17510-16914	13758-11905
	AC083859b	30298-32724	33420-33947	37150-39012
	AC007183b	n.d.	105612-106139	n.d.
	AB062092b	n.d.	60330-60857	n.d.
	AL138663	47920-50396	51025-51621	54762-56457
	AC007155	63893-62482	60937-60341	57550-56301
	AB028614	34361-38022	38650-39243	41493-43343
	AL161506c	129449-130195	131786-132382	134645-135660
	AF160181	n.d.	23554-24150	n.d.
	NM116833	n.d.	970-1524	n.d.
	AF378081	n.d.	25-621	n.d.
	AB026642	15564-12774	12470-11874	8726-6873
	AB073160b	82450-80260	79977-79381	76185-74332
	AB062088	93863-96613	96917-97513	100710-102563
	AB046431b	n.d.	n.d.	n.d.

	AF296831	n.d.	n.d.	n.d.
	NM147974	n.d.	n.d.	n.d.
	AC063973b	101123-101734	102802-103398	105611-105959
	AB046436b	n.d.	89929-90519	n.d.
	AF147259b	25692-23624	22996-22400	n.d.
	AL161506b	n.d.	158078-157482	n.d.
	AC09261a	17563-19421	20022-20618	23659-25452
	AC074111	63611-61184	60556-59951	n.d.
