# Supplementary material for: How Athila retrotransposons survive in the Arabidopsis genome
Source: BMC Genomics. 2008 May 14;9:219. doi: 10.1186/1471-2164-9-219 (PMC2410132; doi:10.1186/1471-2164-9-219)
Supplement: Additional file 3 — Supplementary_table_3 [file 1471-2164-9-219-S3.pdf]

Supplementary Table 3. Positions of the elements shown in Figure 3 (Env tree) in each one of the clones analyzed

	**Env Acc. No.**	*Env position*	**Gag Acc. No.**	*Gag position*
**I**	AB005248	32220-33233	AB005248	26036-27931
	AF272705b	6060-5044	AF272705b	12278-10350
**II**	AC007112.5	17253-18291	AC007112a	13686-15547
	AC026757.12	65159-66213	AC026757.12	61594-62724
	AC009526.4a	82415-81363	AC009526a	86224-84386
	AC009526.4b	73292-72240	AC009526b	77139-75263
	AB046427.1a	43186-42130	AB046427.1	45895-44414
**IIIa**	AF128394.1	8982-7963	AF128394.1	13038-11295
	AC068324.8	36326-35277	AC068324	40206-38397
	AL138654.1	44052-45094	AL138654	39955-41781
	AF272705.1	123689-122644	AF272705.1	127802-125975
	AF147259.1a	7178-8226	AF147259d	3065-4915
	AC051625.6b	32184-33233	AC051625.6b	28107-29864
	AC006267.1	57909-58958	AC006267a	53840-55627
	AC007268.4	58356-59405	AC007268	54217-56048
**IIIa-rec**	AC074111.2	72456-73400	AC074111.2	68286-69875
	AF147264.1c	21044-21984	AF147264a	17231-19060
	AL161506.2f	3389-4329		
	AL161505.2d	196448-197502		
	AF147264.1b	11647-10693	AF147264b	15757-13977
	AL161505.2c	196448-186214		
	AL138643.1	80794-79715	AL138643b	84340-82558
**IIIb**	AL137079.1	45846-46913	AL137079a	39425-41461
	AC006419.3	22824-23767	AC006419b	16642-18676
	AC007125.1b	96343-95278	AC007125.1b	102259-100832
	AL161510.2b	15154-14089		
**Va**	AP002033.1	72243-73300	AP002033.1	68949-70314
	AC006918.8c	75924-74766	AC006918.8a	82277-81426
	AL161505.2a	157540-158616	AL161505c	153648-155629
	AF147262.1a	94448-95524	AF147262.1	90556-91173
	AL391731.1a	37409-38485	AL391731.1a	34440-35498
	AC018928.1	33397-34473	AC018928.1a	28151-29373
	X81801.1	Pelissier etal 1995		
	AP002035.1	30056-28983	AP002035.1	31626-31387
**Va-rec**	AF296829.1a	11596-12672	AB046433c	59651-61654
	AB073163.1	3965-2994	AB073163	10838-8862
**IVb**	AF147263.1b	10824-9756	AF147263a	18674-16713
	AF296831.1c	31091-32136	AB046431a	80931-78877
	AC069557.5	40240-41310	AC069557b	33315-35251
	AC009261.5a	23791-24861	AC009261.5a	17563-19424
	AL161503.2	172932-173996	AL161503	170201-172035
	AF147262.1b	29843-28771	AF147262.1	33797-31947

	AB046431.1a	73936-72866	AB046431a	80931-78877
	AF296831.1b	43966-42896	AF296831.1b	50961-48907
	AB026642.1a	8594-7524	AB026642.1a	15564-13491
	AC007209.5	42154-43224	AC007209.5	35217-37249
	AC007534.2a	97466-98536	AC007534.2a	90480-92545
	AC007534.2b	109678-110739	AC007534a	102696-104762
	AC063973.10a	39615-38545	AC063973.10a	46355-44433
	AB046436.1a	26745-25675	AC063973.10a	46355-44433
	AC083859.1a	37282-38352	AC083859a	30298-32331
	AL138663.1a	54892-55962	AL138663.1a	47920-50003
	AL353871.1	94283-95352	AL353871	87448-89481
**VI**	AB046436.1b	86938-87963	AB046436a	82791-84773
	AC007197.5	67291-68359	AC007197a	63046-65028
	AB046425.1a	25178-26214	AB046425a	21093-23075
	AC069554.4a	71565-70529	AB046425a	21093-23075
	AB046428.1b	50017-51087	AB046428b	45802-47785
	AB073155.1	11376-12323	AB073155d	6994-8995
	AB046433.1a	50430-49360	AB046433e	54716-52767
	AB046426.1a	54012-52942	AB046426b	58164-56183
	AF296828.1	15988-17058	AF296828a	11835-13817
	AC069329.6b	38738-37668	AC069329a	42891-40909
	AB046429.1b	5248-6318	AB046429a	1036-2985
	AB046428.1a	28527-27457	AB046428d	32677-30697
**IVc**	AL138663.1b	32859-33929	AL138663a	32692-34610
	AL161504.2a	40036-41105	AL161504b	35755-37717
	AC007120.5a	22774-21680	AC007120a	26974-24994
	AC073433.9	113126-113893	AC073433	108637-110607
	AF058825.1	66146-67185	AF058825a	61838-63817
	AC069553.6b	17914-18984	AC069553b	13702-15728
	AB046440.1b	27246-28316	AC069553b	13702-15728
	AC083859.1b	77233-76169	AC083859g	80988-78993
	AP002067.2a	2509-1439	AP002067a	6730-4722
	AL161509.2a	55257-54187	AL161509b	59403-57405
	AF262042.1b	47977-46907	AB046433a	43851-41890
	AC006268.1	100730-101799	AC006268b	85184-98503
	AB046428.1c	15121-14051	AB046428.1b	19339-17342
	AF262042.1c	18723-17653	AB046433.1c	14137-12713
	AB046433.1c	10382-9312	AB046433.1c	14137-12713
	AC007125.1a	12515-13583	AC007125a	8267-10264
	AB046431.1b	52158-53228	AB046431b	47835-49809
	AL163975.1	6327-5257	AL163975	10464-8470
	AC007918.2	59964-59017	AC007918a	64256-62265
	AB026642.1b	40647-39577	AB026642.1b	45012-43016
	AP001301.1	64307-65373	AP001301	60121-62118
