# Supplementary material for: How Athila retrotransposons survive in the Arabidopsis genome
Source: BMC Genomics. 2008 May 14;9:219. doi: 10.1186/1471-2164-9-219 (PMC2410132; doi:10.1186/1471-2164-9-219)
Supplement: Additional file 4 — Supplementary_table_4 [file 1471-2164-9-219-S4.pdf]

Supplementary Table 4. Summary of results of PAML tests

II (gag)

		branch dN/dS			
**model**	**lnL**	**a**	**b**	**c**	**d**
M0	-3621.614656	0.2487	0.2487	0.2487	0.2487
M1	-3619.964896	0.3292	0.0001	0.4277	0.2198
M2	-3621.510264	0.2601	0.2601	0.2601	0.2280

test	2ΔL	p-value
M1 vs M0	3.299520	0.347709
M1 vs M2	3.090736	0.213233
M2 vs M0	0.208784	0.647722

II (env)

		branch dN/dS			
**model**	**lnL**	**a**	**b**	**c**	**d**
M0	-1600.859257	0.1959	0.1959	0.1959	0.1959
M1	-1600.743979	0.1371	241.322	0.2312	0.1973
M2	-1600.853042	0.1924	0.1924	0.1924	0.2005

test	2ΔL	p-value
M1 vs M0	0.230556	0.972512
M1 vs M2	0.218126	0.896674
M2 vs M0	0.012430	0.911228

IVb (gag)

		branch dN/dS			
**model**	**lnL**	**a**	**b**	**c**	**d**
M0	-3067.468889	0.2053	0.2053	0.2053	0.2053
M1	-3064.572779	0.0001	947.823	0.2366	0.1160
M2	-3065.518100	0.2442	0.2442	0.2442	0.1169

test	2ΔL	p-value
M1 vs M0	5.792220	0.122169
M1 vs M2	1.890642	0.388555
M2 vs M0	3.901578	0.048241

IVb (RT)

		branch dN/dS			
**model**	**lnL**	**a**	**b**	**c**	**d**
M0	-942.454476	0.1943	0.1943	0.1943	0.1943
M1	-942.454445	0.0001	1.7240	0.1943	0.0001
M2	-942.454460	0.1943	0.1943	0.1943	0.0001

test	2ΔL	p-value
M1 vs M0	0.000062	1.00000
M1 vs M2	0.00003	0.99999
M2 vs M0	0.000032	0.99549

IVb (IN)

		branch dN/dS			
**model**	**lnL**	**a**	**b**	**c**	**d**
M0	-1188.053463	0.0937	0. 0937	0. 0937	0. 0937
M1	-1188.053383	289.2563	0.5901	0.0937	78.8134
M2	-1188.053425	0.0937	0.0937	0.0937	11.6676

test	2ΔL	p-value
M1 vs M0	0.00016	1.00000
M1 vs M2	0.000084	0.99996
M2 vs M0	0.000076	0.99304

IVb (env)

		branch dN/dS			
**model**	**lnL**	**a**	**b**	**c**	**d**
M0	-2402.976208	0.2362	0.2362	0.2362	0.2362
M1	-2399.407933	0.5702	0.5509	0.2028	0.1512
M2	-2402.548959	0.2567	0.2567	0.2567	0.1766

**test**	**2ΔL**	**p-value**
M1 vs M0	7.13655	0.06767
M1 vs M2	6.282052	0.04324
M2 vs M0	0.854498	0.35528

IVc (gag)

		branch dN/dS			
**model**	**lnL**	**a**	**b**	**c**	**d**
M0	-2005.597214	0.1940	0.1940	0.1940	0.1940
M1	-1998.752891	∞	0.0001	0.4693	0.0679
M2	-2000.390860	0.2735	0.2735	0.2735	0.712

**test**	**2ΔL**	**p-value**
M1 vs M0	13.688646	0.003361
M1 vs M2	3.275938	0.194374
M2 vs M0	10.412708	0.001252

IVc (env)

		branch dN/dS			
**model**	**lnL**	**a**	**b**	**c**	**d**
M0	-3757.296537	0.2191	0.2191	0.2191	0.2191
M1	-3754.948183	0.0001	∞	0.3362	0.1834
M2	-3757.081361	0.2314	0.2314	0.2314	0.1893

**test**	**2ΔL**	**p-value**
M1 vs M0	4.696708	0.195401
M1 vs M2	4.266356	0.118460
M2 vs M0	0.430352	0.511816

Va (gag)

		branch dN/dS			
**model**	**lnL**	**a**	**b**	**c**	**d**
M0	-1670.419647	0.3766	0.3766	0.3766	0.3766
M1	-1669.590849	∞	0.0001	0.5072	0.2288
M2	-1669.840244	0.4307	0.4307	0.4307	0.2321

**test**	**2ΔL**	**p-value**
M1 vs M0	1.657596	0.646402
M1 vs M2	0.498790	0.779272
M2 vs M0	1.158806	0.281713

Va (env)

		branch dN/dS			
**model**	**lnL**	**a**	**b**	**c**	**d**
M0	-1990.620157	0.2993	0.2993	0.2993	0.2993
M1	-1986.942536	∞	0.0001	1.0821	0.1261
M2	-1989.311506	0.3482	0.3482	0.3482	0.1434

**test**	**2ΔL**	**p-value**
M1 vs M0	7.355242	0.061397
M1 vs M2	4.737940	0.093577
M2 vs M0	2.617302	0.105704

Va-rec (env)

		branch dN/dS			
**model**	**lnL**	**a**	**b**	**c**	**d**
M0	-1517.240564	0.2133	0.2133	0.2133	0.2133
M1	-1513.940136	0.1634	0.2431	0.3629	∞
M2	-1514.216405	0.1768	0.1768	0.1768	∞

**test**	**2ΔL**	**p-value**
M1 vs M0	6.600856	0.08577
M1 vs M2	0.552538	0.75861
M2 vs M0	6.048318	0.01392

VI (gag)

		branch dN/dS			
**model**	**lnL**	**a**	**b**	**c**	**d**
M0	-3482.052417	0.2100	0.2100	0.2100	0.2100
M1	-3479.941968	6.8492	0.0001	0.2326	0.1160
M2	-3480.846340	0.2347	0.2347	0.2347	0.1161

test	2ΔL	p-value
M1 vs M0	4.220898	0.238578
M1 vs M2	1.808744	0.404796
M2 vs M0	2.412154	0.120397

VI (env)

		branch dN/dS			
**model**	**lnL**	**a**	**b**	**c**	**d**
M0	-1986.034935	0.2054	0.2054	0.2054	0.2054
M1	-1980.047964	0.0001	∞	0.8776	0.0471
M2	-1984.418567	0.2341	0.2341	0.2341	0.0623

test	2ΔL	p-value
M1 vs M0	11.973942	0.007473
M1 vs M2	8.741206	0.012644
M2 vs M0	3.232736	0.072180

VII (gag)

		branch dN/dS			
**model**	**lnL**	**a**	**b**	**c**	**d**
M0	-2939.571110	0.3138	0.3138	0.3138	0.3138
M1	-2934.446446	0.0001	∞	0.9998	0.2135
M2	-2939.040850	0.3415	0.3415	0.3415	0.2326

test	2ΔL	p-value
M1 vs M0	10.249328	0.016561
M1 vs M2	9.188808	0.010108
M2 vs M0	1.060520	0.303097

VII (RT)

		branch dN/dS			
**model**	**lnL**	**a**	**b**	**c**	**d**
M0	-1076.314401	0.3155	0.3155	0.3155	0.3155
M1	-1067.106586	0.0001	∞	1.1605	0.0001
M2	-1069.746529	0.4563	0.4563	0.4563	0.0001

test	2ΔL	p-value
M1 vs M0	18.4153	0.00036
M1 vs M2	5.279886	0.07137
M2 vs M0	13.135744	0.00029
